# Response Surface Methodology to Optimize the Extraction of Carotenoids from Horticultural By-Products—A Systematic Review

**DOI:** 10.3390/foods12244456

**Published:** 2023-12-12

**Authors:** Marina Cano-Lamadrid, Lorena Martínez-Zamora, Laleh Mozafari, María Carmen Bueso, Mathieu Kessler, Francisco Artés-Hernández

**Affiliations:** 1 Postharvest and Refrigeration Group, Department of Agricultural Engineering, Institute of Plant Biotechnology, Universidad Politécnica de Cartagena, 30203 Cartagena, Murcia, Spain; marina.cano@upct.es (M.C.-L.); lorena.martinez@upct.es (L.M.-Z.); laleh.mozafari@edu.upct.es (L.M.); 2 Department of Food Technology, Nutrition and Food Science, Faculty of Veterinary Sciences, University of Murcia, 30071 Espinardo, Murcia, Spain; 3 Department of Applied Mathematics and Statistics, Universidad Politécnica de Cartagena, 30202 Cartagena, Murcia, Spain; mcarmen.bueso@upct.es (M.C.B.); mathieu.kessler@upct.es (M.K.)

**Keywords:** by-products revalorization, food loss, food waste, green technology, circular economy, RSM

## Abstract

Response Surface Methodology (RSM) is a widely used mathematical tool for process optimization, setting their main factorial variables. The current research analyzes and summarizes the current knowledge about the RSM in the extraction of carotenoids from fruit and vegetable by-products, following a systematic review protocol (Prisma 2020 Statement). After an identification of manuscripts in Web of Science (September 2023) using inclusion search terms (“carotenoids”, “extraction”, “response-surface methodology”, “ultrasound”, “microwave” and “enzyme”), they were screened by titles and abstracts. Finally, 29 manuscripts were selected according to the PRISMA methodology (an evidence-based minimum set of items for reporting in systematic reviews), then, 16 questions related to the quality criteria developed by authors were applied. All studies were classified as having an acceptable level of quality criteria (≤50% “yes answers”), with four of them reaching a moderate level (>50 to ≤70% “yes answers”). No studies were cataloged as complete (>70% “yes answers”). Most studies are mainly focused on ultrasound-assisted extraction, which has been widely developed compared to microwave or enzymatic-assisted extractions. Most evidence shows that it is important to provide information when RSM is applied, such as the rationale for selecting a particular design, the specification of input variables and their potential levels, a discussion on the statistical model’s validity, and an explanation of the optimization procedure. In addition, the principles of open science, specifically data availability, should be included in future scientific manuscripts related to RSM and revalorization.

## 1. A Short Overview of Response Surface Methodology Applied in By-Products Revalorization Area

**Figure 1:**
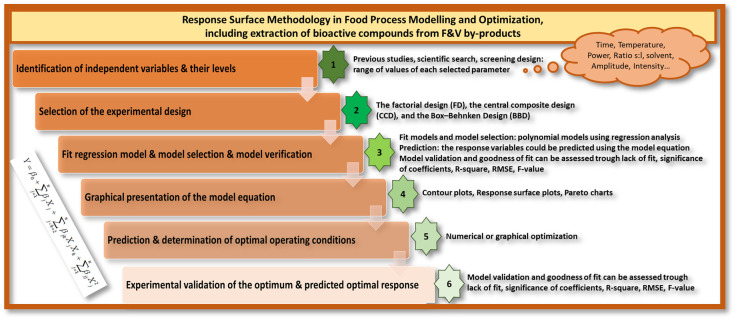
Steps for properly implementing Response Surface Methodology in Food Process Modelling and Optimization.

Response Surface Methodology (RSM) is a widely used mathematical tool for processes optimization, setting their main factorial variables. Improving system performance and increasing process efficiency without increasing cost and time is the most important objective of food processing. The main purpose of “optimization” is to find a combination of factors with the best response for a system. Once upon a time, the effect of changes in one variable on a response was investigated in a food process with all other variables held constant, without considering the combined effect of the variables. Also, it increases the number of experiments needed to carry out the research, which leads to an increase in cost and time. To solve this problem, multivariate statistical methods can be used for optimization research. Therefore, RSM is commonly used in food processing for experimental design and optimization. RSM involves fitting a polynomial model to data that should represent the behavior of a data set for the purpose of making statistical predictions, determining the design factor settings to improve/optimize the performance or response of a process. It combines design of experiments, regression analysis and optimization methods in a general-purpose strategy to optimize the expected value of a stochastic response [1]. Consequently, when responses are affected by several inputs, this tool can be used to optimize, design, develop and improve processes. The modelling and optimization using the RSM approach, involves several steps to be performed as Figure 1 shows. In the following sections, more detailed information of each step is included [2].

It is common to perform many experiments to improve the extraction process of bioactive compounds of interest, such as optimizing the yield in an extraction process [3]. The analysis of the obtained results is usually performed using tools that require many tests, increasing the economic cost of the development of an optimized process. To solve this problem, an effective tool is the use of RSM, since it allows an experimental design with a reduced number of tests [4]. 

There is an extensive literature on the extraction of bioactive compounds from horticultural by-products using RSM, but not many of them use RSM as a target to draw optimized conclusions. Also, the inputs, levels, outputs, experimental design, and graphical presentation, among other specifications, vary depending on the research. This work is a systematic review to find out the specifications of RSM-related investigations used by researchers in the extraction of carotenoids from horticultural by-products. Following the proposed quality criteria, the aim was to assess the completeness and clarity (detailed description of the experimental design, justification of the selection of a particular design, specification of the input variables and their potential levels, discussion of the validity of the statistical model, and explanation of the optimization procedure) of the manuscripts that already applied RSM in this subject. The novelty of this new contribution and the discussion made by the authors is that this is the first systematic review related to the topic, presenting a detailed investigation of previous research to identify, categorize, analyze, and report the aggregate evidence on carotenoid extraction from horticultural by-products.

### 1.1. Steps for Properly Implementing Experimental Response Surface Methodology

#### 1.1.1. Identification of Inputs and Their Levels

Ultrasound (USAE), microwave (MWAE) and enzyme-assisted extraction (EAE) are the most used extraction techniques for health promoting compounds from horticultural commodities [3]. The independent variables (inputs) must be first established and their range of values for operation must be well-known. It is common to perform a screening design to select the important main effects and discard the less important ones, thus selecting inputs and their ranges. Researchers/professionals can also rely on the scientific literature to design the optimization. The limitations of the equipment used must be known as it will affect the ranges of some variables. The most important inputs for USAE, MWAE, and EAE are deeply detailed in a previous study by the same authors [3]. Once the inputs have been selected, the type of RSM design and thus the number of levels must be selected based on previous studies. This step within the RSM design is detailly discussed in the next section. 

#### 1.1.2. Selection of the Experimental Response Surface Methodology Design

Depending on the objectives of the experiment and the number of factors investigated, a design type is selected. Among the RSM designs, the most common ones for horticultural by-product revalorization are subsequently detailed.

***Factorial design***

A factorial design allows to study the effects that several factors can have on a response. When conducting an experiment, varying the levels of all factors at the same time instead of one at a time allows to infer about possible interactions between factors. The three-level design, which is written as a 3^n^ factorial design, is subsequently explained. It means that n factors are considered, and each factor at 3 levels: low, intermediate, and high levels; expressed as −1, 0, +1. A third level for a continuous factor facilitates investigation of a quadratic relationship between the response and each of the factors. However, the full factorial design (FFD) requires many experimental runs for a precise modelling of quadratic relation, when the number of factors is greater than 2. Other designs have been proposed to reduce the number of required runs, taking advantage of the quadratic structure of the relationship. An example is a fractional factorial design (FrFD), where researchers conduct only a selected fraction of the runs in the FFD. In the case when the selected fraction if 50%, the design is called as half-fractional factorial design (HFD). Fractional factorial designs are a good choice when resources are limited or the number of factors in the design is large because they use fewer runs than the FFD [5]. To reduce the number of experiments, other RSM design can be carried out such as central composite design (CCD) and Box–Behnken design (BBD), which are subsequently detailed [2].

***Central composite design***

A Box-Wilson central composite design (CCD), commonly called ‘a central composite design’, contains an embedded factorial or fractional factorial design with center points that is augmented with a group of “star/axial points” that allow estimation of curvature. If the distance from the center (0) of the design space to a factorial point is ±1 unit for each factor, the distance from the center of the design space to a star point is |α| > 1. The precise value of α depends on certain properties desired for the design and on the number of factors involved. Similarly, the number of center points that the design needs is to contain also depends on certain properties required for the design. A CCD always contains twice as many star points as there are factors in the design. The star points represent new extreme values (low and high) for each factor [2]. Among CCD types, there are different specific designs. In rotable designs (CCRD), for example, the variance in the predicted response depends only on its distance from the central point of the design, and the value of the parameter α required is computed as α = 2^(n/4)^. In the face-CCD, the design points are placed at the midpoints of the edges (face-center points) and at the center of the design space [6].

***Box–Behnken design***

The Box–Behnken design (BBD) is an independent quadratic design which does not contain an embedded factorial or fractional factorial design. In this design the treatment combinations are at the midpoints of edges of the process space and at the center. These designs are rotatable (or near rotatable) and require 3 levels of each factor: −1, 0 and +1 [7]. The designs have limited capability for orthogonal blocking compared to the central composite designs [2].

#### 1.1.3. Selection of a Regression Model and Prediction and Verification of Model Equation

After running the selected designed experiment, different regression models can be fitted to the data. The estimation of the model coefficients uses the least squares criterion, which minimizes the sum of squared differences between the observed and predicted values. The statistical model assumes normality, independence, and homogeneity of variance of the errors, which makes possible to test for significance of coefficients, obtain confidence intervals and analysis of variance tables. The choice of model order should be guided by a balance between model complexity and model fit to the data surface. In practice, it is common to start with a lower-order model (e.g., linear or quadratic) and then assess model adequacy through diagnostics, such as residual analysis and statistical tests (e.g., significance of the terms and analysis of variance), goodness fit criteria (e.g., R-squared and adjusted R-squared), and model selection criteria (e.g., AIC and BIC). If the lower-order model does not adequately represent the system, higher-order models can be considered [1].

Ultimately, the choice of model order in RSM should be based on a combination of statistical analysis, domain knowledge, and the goals of the study. To validate the assumptions of linearity, homoscedasticity (homogeneity of variance), and normality for the error in the regression model, an analysis of the residuals (differences between observed and predicted values) should be conducted, including both statistical tests and diagnostic plots (such as residual plots where residuals are plotted against the fitted values and normality probability plots). If these assumptions are significantly violated, it may be convenient to consider transforming the response variable (e.g., using a logarithmic transformation). Once the model is validated, the response variable can be predicted for different combinations of the factor levels. Plotting observed values against the model’s predicted values is useful to gain confidence in the predictive capacity of the model [1].

#### 1.1.4. Graphical Presentation of the Model Equation

The most common graphical presentations of the data after RSM in Food Technology are: 

*Surface response plots:* 3D surface graphs are used to know the values of the responses depending on the variables studied. It is a useful three-dimensional surface graph that contains a series of elements detailed below: the X and Y axes where the studied variables are located and a continuous surface that represents the response values on the Z axis. The different combinations show a series of peaks that correspond to the maximum values of the response and valleys corresponding to the minimum values [8].

*Contour plots:* The contour graph is a two-dimensional graph that is used to relate the two independent variables, X and Y, and the response, Z. The graph shows the different values of the response depending on the various combinations of the variables X and Y that correspond to the X and Y axes. Z values are represented in contour lines and bands [8]. 

*Pareto charts:* It is a bar graph in which the “y” axis details the frequency (left side), and the “z” axis includes the percentage (right side). Each of the factors included are ordered descending by frequency on the x-axis. If the line reaches ≥80%, it is concluded that all the factors previously added represent 20% of the causes (it is called the 80/20 rule, being an approximate guide to typical distributions based on the Pareto principle). Therefore, the Pareto diagram highlights that the effects of the contributing factors that lead to a specific outcome are not equal [9].

The *Principal Component Analysis* (PCA) plot, although it is not a typical graphical presentation of an optimization, is hereby included because some of the selected articles in this review used it. PCA is a dimensionality reduction method as it projects observations from a p-dimensional space (p variables) to a k-dimensional space (where k < p) to conserve the maximum amount of information contained in the data (information is measured here through the total variance of the dataset) [10].

#### 1.1.5. Prediction and Determination of Optimal Operating Conditions

After validating the regression model to accurately represent the response surface, the established model is used to predict the response for any combination of factor levels. The visualization of the response surface through 3D surface plots or contour plots helps determining the conditions that optimize (either maximize or minimize) the response variable. Optimization algorithms such as the gradient ascent/descent or other iterative methods can be employed to find the optimal conditions within the experimental domain. Many statistical software includes functions for the numerical determination of optimum. Additional experiments can be performed to verify the identified optimal operating conditions.

## 2. Importance of Carotenoids from Horticultural By-Products

Historically, natural pigments have been widely used in medicine, the food industry, cosmetics, the fashion industry, and furniture, among other processes. In addition, due to their chemical structure, these pigments, with known pharmacological properties, are of interest to scientists, because of their extensively reported health-promoting benefits as antioxidant, anti-inflammatory, and antimicrobial [11]. The most accumulated pigments in nature are chlorophylls, carotenoids, anthocyanins, betacyanins, and flavonoids, and as they act as photoreceptors in plants, they are mainly concentrated in leaves, fruit peels, or flowers, in other words, the non-edible parts of the plants [11]. In this sense, carotenoids are primarily synthesized by plants and algae, imparting the distinctive yellow, orange, and red hues to various fruits and vegetables [12].

Over a thousand different carotenoid species have been identified and grouped into two primary categories; most of these originated in the plant kingdom: xanthophylls, which contain oxygen in their chemical composition, and carotenes, which consist of hydrocarbon chains without oxygen. In a general biochemical sense, carotenoids share a polyene chain structure with multiple double bonds and potential ring structures at their ends. This structural feature underpins their biological functions, particularly their capacity to act as electron donors within the molecule, which forms the basis of their antioxidant activity [13]. Notably, when plant carotenoids function as light absorbers, they play pivotal roles in initiating photosynthesis reactions, providing protection against environmental stressors, influencing plant coloration, and participating in cell signaling processes [14]. 

Among carotenoids rich foods, tomatoes, and their derivatives, such as ketchup, juices, or sauces, are the widest source of lycopene, although the richest food on this compound is Gac fruit arils, derived from *Momordica cochinchinensis* (Lour.) [15]. Also, watermelon, papaya, and guava contain high concentrations of this compound. β-Carotene is the dominant carotenoid in carrots, sweet potatoes, capsicum pods, and leafy green vegetables [16]. Green leafy vegetables are rich in lutein and β-carotene, followed by neoxanthin and violaxanthin. Among green leafy vegetables, lactucaxanthin is uniquely present in lettuce (*Lactuca sativa* L.), with a high concentration in romaine lettuce (cv. Super caesar red). Furthermore, corn (*Zea mays* L.) seeds and egg yolk serve as good sources of lutein and zeaxanthin [16]. Given that corn makes up more than 50% of laying-hen diets [16], it contributes to the vibrant yellow-orange color of egg yolk, attributed to lutein (0.714 mg/100 g). Citrus fruits, persimmons, peaches, papayas, and capsicum pods are significant sources of β-cryptoxanthin in the diet [16]. In most fruits, xanthophylls such as neoxanthin, lutein, zeaxanthin, and β-cryptoxanthin are primarily found in esterified forms (comprising around 50–99% of total xanthophylls) [16]. Red paprika (*Capsicum annuum* L.; bell pepper) pods from various cultivars contain total carotenoids contents, with capsanthin accounting for approximately 75% of the content, except in cv. Mini Goggal Red, where zeaxanthin makes up 96% of the total carotenoids [17].

In this context, discarded peels, seeds, roots, and leaves from the food industry serve as the primary reservoirs for carotenoid extraction. Notably, these by-products contain substantial levels of water, oxygen, and nitrogen, which can potentially pose significant contamination concerns. Consequently, given their rich nutrient content, there is an opportunity to recover and repurpose these food wastes. Traditionally, methods employed for extracting plant carotenoids encompass the use of solvents, fermentation, enzymatic processes, and emerging technologies such as microwaves, ultrasonic treatments, cold plasma, and supercritical fluids. After extraction, to obtain pure carotenoid compounds, these extracts undergo purification through solid-phase extraction, followed by separation using chromatographic techniques [18]. In recent years, green technologies such as USAE, MWAE, and EAE have seen significant advancement in their application for carotenoid extraction. However, conventional approaches, including thermal extraction and the use of polar solvents and oils, continue to be widely utilized for isolating carotenoids from primary food sources.

In this scenario, carotenoid extracts have a wide range of applications in the food industry due to their vibrant colors, antioxidant properties, and potential health benefits. Common uses of carotenoid extracts in the food industry include: (i) food coloring (as pigment, carotenoids can impart shades ranging from yellow to red and are utilized in a diverse array of food items, including beverages, confectionery, baked goods, dairy products, sauces, and dressings); (ii) nutraceuticals that offer health benefits beyond basic nutrition; (iii) preservatives due to their antioxidant capacity that help to extend the shelf-life of a great variety of commodities.

## 3. Methodology

The methodology used was based on the updated PRISMA guidelines for a systematic review (Table S1). The following sub-sections provide the details included in the checklist for reporting this research [19].

### 3.1. Search and Eligibility Criteria

WoS was used as a scientific database for searching documents (15 September 2023). The terms “carotenoids”, “extraction”, and “response-surface methodology” were used as search words, and the following items were also used: “ultrasound” OR “microwave” OR “enzyme”. From the bibliography found and described in the raw analysis, the inclusion criterion for our systematic review was ‘original studies related to horticultural by-products included in JCR-SCI journals’. The exclusion criteria were books, reviews, and experiments carried out with horticultural commodities but not extracted from by-products.

### 3.2. Data Synthesis: PRISMA Flow Diagram

**Figure 2:**
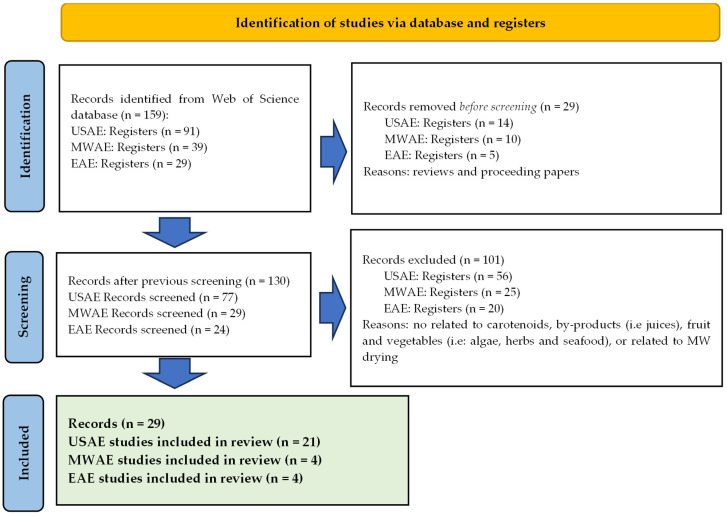
Flow diagram describing the study selection process of the scientific literature page.

The title and abstracts of the documents found were analyzed and classified depending on their significant interest using Microsoft Excel for the data curation. First, papers not focused on the studied field were excluded from the identification step. The assessment of each record was performed by one reviewer (M.C.-L.). It is known that a single screening is an efficient use of time and resources, but there is a higher risk of missing relevant studies. Then the potential papers were submitted to exhaustive analysis in the screening step, where all the papers were checked in search of inclusion criteria (Figure 1). In this step, the assessment of selected records was performed by two reviewers (L.M.-Z. and M.C.-L.). It means that this approach may be more reliable than single screening, but at the expense of increased reviewer time, given the time needed to resolve discrepancies. The researchers independently screened the titles and abstracts of all articles retrieved. In cases of disagreement, consensus on which articles to screen in full text was reached through discussion. The PRISMA flow diagram [19] and the obtained results of the systematic review are shown in Figure 2.

### 3.3. Data Collection Process and Quality Criteria

The potential scientific papers were subjected to a comprehensive analysis, in which all the manuscripts were checked for inclusion of quality criteria. The data items collected followed the proposed questions. No assumptions were made about any missing or unclear information from the studies. A total of 16 questions were used as quality criteria based on previous literature [1,2] and the “know-how” of the research team (Table 1). These questions were also used to prevent bias in the conclusions. Three authors of the present review used them to extract data from eligible studies, which were entered into Excel software, triple checking this for accuracy. The extracted data were compared, with any discrepancies being resolved through discussion among all authors. Possible sources of bias (the main limitations) of our study include (i) language, (ii) the small number of articles included, (iii) chosen databases, (iv) inclusion and exclusion criteria, (v) the selected criteria questions, (vi) fruit and vegetable scope (excluding algae, herbs, and seafood), and (vii) the impact of missing data.

Each question was evaluated as “yes”, “no”, or “unclear” and the analysis of the answers was carried out as previously reported [20]. The frequency of “yes” answers for each item was used to determine the quality and reproducibility of the RSM and evidence regarding the extraction of carotenoids from horticultural by-products. The risk of bias in the selected works was rated in three categories, expressing the completeness of the description of the experimental design and data analysis: complete (>70% “yes” answers), moderate (>50 to ≤70% “yes” answers), and acceptable (≤50% “yes” answers).

The following specifications were included as a collection of information related to each of the questions: type of design, number and type of input factors and responses, number of levels, purpose, number of experiments; source of the information that led to the factors’ specification types of RSM quality indicators. The selected design for the experiment was also registered: (i) factorial design (FD): full and/or fractional factorial design (FFD/FrFD) (number of factors); (ii) central composite design (CCD) (requires to be able to set five levels per factor); or (iii) Box–Behnken design (BBD) (only three possible levels per factor). Regarding question Q13, the authors asked two sub-questions: (a) Was an optimum value found within the experimental domain? and, (b) Was no optimum value was found within the experimental domain, but a direction of search is indicated?

## 4. Application of RSM to Carotenoids Extraction from Horticultural By-Products

**Figure 3:**
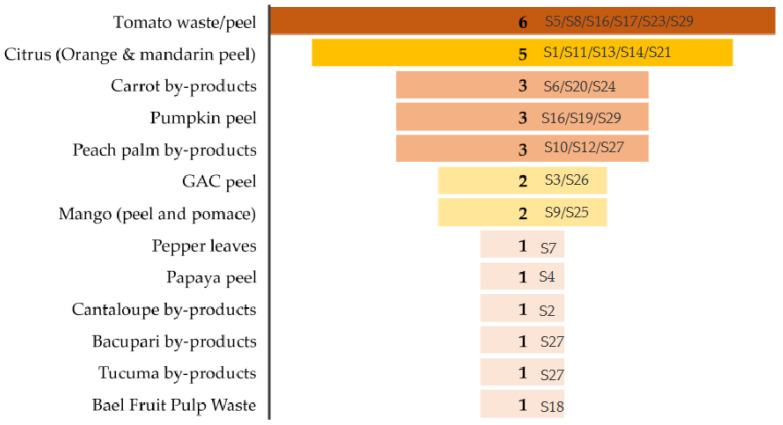
Horticultural by-products used for carotenoid extraction by RSM (S1–S29: scientific manuscripts, references included in Table 2).

Following the selection of manuscripts by the PRISMA method, Figure 3 shows the different types of by-products used for carotenoid extraction using the RSM. The largest number of studies were found to be related to industrial tomato pomace/waste/peel, followed by citrus (sum of articles related to orange and mandarin peel) and carrot by-products. This observation was consistent with a previous study that concluded that tomato waste and by-products are the primary raw materials for lycopene production [21].

### Quality Criteria of the Selected Papers and Breakdown of the Quality Criteria

Following the questions established in Section 3.2, Table 2 shows the response of each of the studies included in this bibliographic review. Three of these studies have more than one well-differentiated section, including more than one RSM study, and that is why information is individually collected.

**Figure 4:**
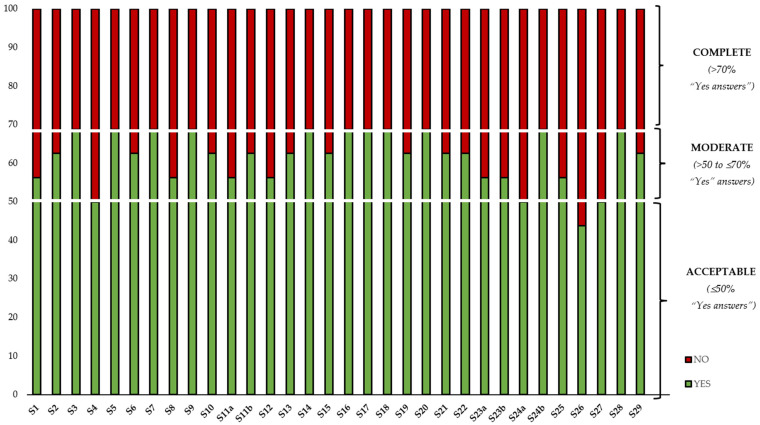
Evaluation of the quality of the reviewed works.

Besides Table 2, Figure 4 shows the percentage of total responses in each study (and the inclusion of the three established levels of quality criteria). It should be noted that all the included studies were evaluated by peer reviewers, which is why they have been published based on the journal’s criteria. These levels established by the authors of this work are levels based on the RSM. All studies were classified as having an acceptable level of quality criteria (≤50% “yes answers”), with four of them reaching the moderate level of quality criteria (>50 to ≤70% “yes” answers) [22,23,24,25]. No studies were cataloged as complete (Table 2 and Figure 4). 

**Figure 5:**
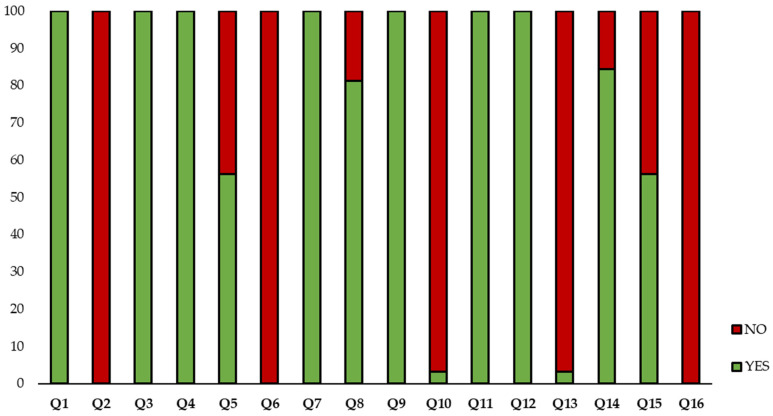
Percentage of affirmative and negative responses from all studies for each question.

On the other hand, Figure 5 shows the percentage of affirmative and negative responses of all studies of each question. It must be specified that authors of this work have considered that the total number of studies was 32, not 29 as indicated in the PRISMA graph. The reason is that three of the studies included more than one RSM design and information was individually collected. It should be noted that none of the included studies reported information related to three of the sixteen questions (Q2, Q6, and Q16). Additionally, two of the criteria (Q10 and Q13) only included information in one of the studies [26]. On the contrary, questions Q1, Q3, Q4, Q7, Q9, Q11, and Q12 were answered by 100% of the included studies. In the following lines, detailed information of each question was discussed.

**Table 2:** Evaluation of the quality of the reviewed publications.

Technique	Study	Q1	Q2	Q3	Q4	Q5	Q6	Q7	Q8	Q9	Q10	Q11	Q12	Q13	Q14	Q15	Q16	Ref.
USAE	**S1**	YES	NO	YES	YES	NO	NO	YES	NO	YES	NO	YES	YES	NO	YES	YES	NO	[27]
	**S2**	YES	NO	YES	YES	YES	NO	YES	NO	YES	NO	YES	YES	NO	YES	YES	NO	[28]
	**S3**	YES	NO	YES	YES	YES	NO	YES	YES	YES	NO	YES	YES	NO	YES	YES	NO	[29]
	**S4**	YES	NO	YES	YES	NO	NO	YES	YES	YES	NO	YES	YES	NO	NO	NO	NO	[22]
	**S5**	YES	NO	YES	YES	YES	NO	YES	YES	YES	NO	YES	YES	NO	YES	YES	NO	[30]
	**S6**	YES	NO	YES	YES	YES	NO	YES	YES	YES	NO	YES	YES	NO	YES	NO	NO	[31]
	**S7**	YES	NO	YES	YES	YES	NO	YES	YES	YES	NO	YES	YES	NO	YES	YES	NO	[32]
	**S8**	YES	NO	YES	YES	YES	NO	YES	NO	YES	NO	YES	YES	NO	YES	NO	NO	[33]
	**S9**	YES	NO	YES	YES	YES	NO	YES	YES	YES	NO	YES	YES	NO	YES	YES	NO	[34]
	**S10**	YES	NO	YES	YES	NO	NO	YES	YES	YES	NO	YES	YES	NO	YES	YES	NO	[35]
	**S11a**	YES	NO	YES	YES	YES	NO	YES	YES	YES	NO	YES	YES	NO	NO	NO	NO	[36]
	**S11b**	YES	NO	YES	YES	YES	NO	YES	YES	YES	NO	YES	YES	NO	YES	NO	NO	[36]
	**S12**	YES	NO	YES	YES	NO	NO	YES	YES	YES	NO	YES	YES	NO	YES	NO	NO	[37]
	**S13**	YES	NO	YES	YES	YES	NO	YES	YES	YES	NO	YES	YES	NO	YES	NO	NO	[38]
	**S14**	YES	NO	YES	YES	YES	NO	YES	YES	YES	NO	YES	YES	NO	YES	YES	NO	[39]
	**S15**	YES	NO	YES	YES	NO	NO	YES	YES	YES	NO	YES	YES	NO	YES	YES	NO	[40]
	**S16**	YES	NO	YES	YES	YES	NO	YES	YES	YES	NO	YES	YES	NO	YES	YES	NO	[41]
	**S17**	YES	NO	YES	YES	YES	NO	YES	YES	YES	NO	YES	YES	NO	YES	YES	NO	[42]
	**S18**	YES	NO	YES	YES	YES	NO	YES	YES	YES	NO	YES	YES	NO	YES	YES	NO	[43]
	**S19**	YES	NO	YES	YES	NO	NO	YES	YES	YES	NO	YES	YES	NO	YES	YES	NO	[44]
	**S20**	YES	NO	YES	YES	YES	NO	YES	YES	YES	NO	YES	YES	NO	YES	YES	NO	[45]
	**S21**	YES	NO	YES	YES	NO	NO	YES	YES	YES	NO	YES	YES	NO	YES	YES	NO	[46]
MWAE	**S22**	YES	NO	YES	YES	NO	NO	YES	YES	YES	NO	YES	YES	NO	YES	YES	NO	[47]
	**S23a**	YES	NO	YES	YES	NO	NO	YES	YES	YES	NO	YES	YES	NO	YES	NO	NO	[48]
	**S23b**	YES	NO	YES	YES	NO	NO	YES	YES	YES	NO	YES	YES	NO	YES	NO	NO	[48]
	**S24a**	YES	NO	YES	YES	YES	NO	YES	NO	YES	NO	YES	YES	NO	NO	NO	NO	[23]
	**S24b**	YES	NO	YES	YES	YES	NO	YES	YES	YES	NO	YES	YES	NO	YES	YES	NO	[23]
	**S25**	YES	NO	YES	YES	YES	NO	YES	YES	YES	NO	YES	YES	NO	NO	NO	NO	[24]
EAE	**S26**	YES	NO	YES	YES	NO	NO	YES	NO	YES	NO	YES	YES	NO	NO	NO	NO	[49]
	**S27**	YES	NO	YES	YES	NO	NO	YES	NO	YES	NO	YES	YES	NO	YES	NO	NO	[25]
	**S28**	YES	NO	YES	YES	NO	NO	YES	YES	YES	YES	YES	YES	YES	YES	NO	NO	[26]
	**S29**	YES	NO	YES	YES	NO	NO	YES	YES	YES	NO	YES	YES	NO	YES	YES	NO	[50]

As expected, the answer to question Q1 was affirmative in all the selected studies, as this was one of the objectives. The authors of this review collected information on which type of RSM design each used. The most used design in the selected articles was CCD, followed by BBD (Table 3). In terms of design types, 53% of the included studies used the CCD, 23% of them were CCRD, and 5.9% were face-centered. On the other hand, 31% of the studies were BBD, followed by FD, with 80% of the FD studies being FrFD. In addition, there are several studies in which screening experimental designs are included to select the most appropriate variables and ranges [27,36]. After the collection of information related to the design of the selected articles, it can be observed that the most used software for the design of experiments (DOE) was Design Expert software (42%), followed by JMP^®^ Statistical Software (16%) and Statistical Software (10%) in their different versions and updates.

Regarding Q2, although it is specified that they were based on previous studies, it does not refer to the exact reason for the selection of the design. As described above, the RSM designs aims to reduce the number of total samples; in some articles, it was just mentioned that the reason for selecting a specific design was “minimizing number of experiments”. Therefore, the answer to question Q2 was negative in all selected articles. This information is very useful for readers of the articles as it helps to understand the design and to be able to apply that knowledge for future experiments. 

As expected, 100% of the studies included the inputs and responses (Q3) and the number of factor levels (Q4). Table 4 shows the number of inputs and responses in each of the studies. It should be noted that within the responses, the carotenoid content is included. Some of them express it as total carotenoids, either by spectrophotometric methods or by liquid chromatography. Also, some studies were focused on monitoring one or more individual carotenoids, such as lycopene or beta-carotene, identified and quantified by liquid chromatography. As mentioned in Section 1.1.1, the most common inputs used were time (I1), temperature (I2), ratio of solid:liquid (I3), and solvent (I4). Also, it is essential to highlight that some inputs were specific to the extraction technique used (USAE, MWAE, or EAE) and were included in almost 100% of the studies. For instance, microwave power was just included in MWAE studies, or enzyme concentration was just included in the articles focused on EAE. As to the number of factor levels, it is well known that at least five levels per factor are required in CCD and only three levels per factor are required in BBD [51]. Table 4 shows the number of levels per factor in each study, and it is essential to highlight that almost 30% of the studies in which CCD was selected as RSM design indicated three levels of each factor. It is necessary to note that three of these studies indicated three levels (−1, 0, +1) in the text, but in the tables of the combinations, five levels were detailed [22,32,45]. In addition, information related to the number of axial points was also included in some studies in which CCD was used. Also, it is important to highlight that the reason for the selection of the inputs and the levels of each of the factors (Q5) were not included in all the selected articles; any type of information justifying the selection was included in 41% of them. It should be noted that in the articles that included the reason, 100% indicated that they were based on previous studies. There are no studies that indicate that they were based on scientific literature. It is also reported in some documents that the selection was also made due to the limitations of the equipment [31] and the objective of the study [31]. For instance, temperatures higher than 60 °C degrade bioactive compounds. 

As to question Q6 (Do the authors report a transformation of the response data?), no affirmative response was obtained. In cases where the residual analysis raises some issues about the statistical assumptions associated with the regression model (linearity, homoscedasticity, and normality of errors), applying transformations to the response variable (e.g., using the square root or logarithmic function) may lead to residuals that are more normally distributed and homoscedastic, improving the accuracy of statistical inferences derived from the fitted model. 

As expected, the number of experiments needed to develop a good RSM (Q7) was included in all studies, even though no relevant information related to the number of experiments was included in one of them [33]. Table 4 shows the number of experiments for each study. The wide range of values, from 8–31, is striking. It is also worth mentioning that the number of centers ranged from 2–7. This variability depends on the choice of inputs, the choice of design, and the number of levels in each factor set. The information included in this section is essential to knowing the complete experimental design, which is not included completely in some of the selected articles.

An affirmative response was observed in a high percentage of the studies concerning Q8 (Figure 4). In some of them, it is specified in a column in the tables where “run order” was included, while in others, despite answering that the order carried out was included, it was not clear if it was the order carried out in the experimental part or the order in which the data were curated. It should be noted that in two of the studies, in addition to the randomization of the samples showing the specific order in a table, it was indicated that “*The six center point runs were not randomized, but evenly interspersed among the other experimental points, since they provide a measure of process stability and allow estimating inherent variability of the process and therefore must be regularly verified throughout the experiment*” [41,42]. The rest of the documents in which the question has been negatively answered is because they did not show any type of evidence regarding the order of the experiments. Other authors indicated “random”, but it is not enough information to know the established order [28]. More detailed information related to “run order” should be included in future experiments; this information ensures the reproducibility of the study. 

Predictably, graphical presentation (Q9) was included in 100% of the studies, but not all included the same types of graphics. From surface response plots to PCA plots, including contour plots and others. This information is detailed in Table 3, and it should be specified that, as expected, >80% of the studies used response surface plots, while 18% of them were supported by contour plots. On the other hand, two of the included studies used Pareto plots, and one of them used common two-dimensional plots. Furthermore, the PCA plot was included in two of the studies but supported by response surface plots.

In contrast, regarding the question focused on the availability of open data (Q10), none of them includes such a possibility, with only one indicating, “*The datasets generated during and/or analyzed during the current study are available*” [26]. Another study indicated in their Data Availability Statement, “*Data is contained within the article*”, but no data was included in the manuscript. Therefore, this manuscript was not included as an affirmative response to Q10 [32].

In the question on indicators (Q11), a search for the following indicators was carried out: R-squared, adjusted R-squared, lack of fit, F-value, *p*-value, ANOVA results, and pos-hoc results. It can be concluded that 100% of the articles showed at least one of these indicators. It should be noted that >40% showed the indicators mentioned. According to expectation, the effect of each factor on the response (Q12) was included in 100% of the studies. Although it seems an obvious question, it was necessary to include it to obtain a complete quality criterion in RSM studies. 

As to Q13 (Does the paper mention whether the statistical hypothesis of the model was checked?), it can be detailed that the authors of this review search for information such as “error normality”, “homoscedasticity” (in diagnostic plots or test including residuals), and “Gauss bell” in the methodology and results/discussion section. It should be emphasized that this information was just included in graphs in one study (normal plot of Residuals and Residual vs. Predicted), the same article in which “data availability” was included [26]. 

Q14 and Q15 are related since the first of them focuses on whether an extraction optimization is included and the second is whether a validation of the optimal condition has been carried out at the end of the study. It is essential to highlight that optimization was included in more than 80% of the studies. Theoretically, where the mathematical models were considered predictive and significant, the optimized conditions were experimentally verified (validation) to compare the predicted and experimental values to check the reliability of the predictive models (the data can be presented in Text, Tables and/or Plots of the actual vs. predicted response). Among the manuscripts with the optimization step included, 67% included the validation (predicted value vs. actual result without significant differences), but the rest of them did not include information indicating that the optimization of the process was not yet achieved because an optimized region was not observed, except in one study [49]. 

Finally, none of the studies included information related to the code for data analysis (Q16), which is linked with Q10 (data availability). Open Science is a movement promoted by the OECD countries and driven by the European Commission that advocates for free access by citizens to the results of scientific research—data, resources, results, thoughts, as well as the results and discoveries of scientific research—to be accessible universally and without restrictions. Open science involves several items, such as open access publishing or open peer review, but also data availability and data code availability. Therefore, as expected, following the review and compilation of information related to open science, a point for improvement was identified in this regard.

Sustainable solvents are a topic of growing interest in both the research community and the industry, including the fields of horticulture and agriculture, due to a growing awareness of the impact of solvents on pollution, energy usage, and contributions to air quality and climate change. Green chemistry (described as the design of chemical products and processes to eliminate or reduce the use and generation of hazardous substances, following 12 principles) represents a new paradigm in agriculture as it serves as a driving force for sustainable agricultural development (the 2030 Agenda’s Sustainable Development Goals). To counter these issues, a range of greener or more sustainable solvents have been proposed and developed over the past three decades [52]. More green and eco-friendly solvents are more desirable than conventional ones for the extraction of bioactive compounds from horticultural by-products [53]. Therefore, additional information related to what types of solvents have been used in the selected studies, both in USAE and in MWAE, is detailed below. The information related to EAE has not been incorporated since it works with the enzymes’ own solutions. It is striking that several studies used vegetable oils (mainly sunflower oils [22,37,38], but flaxseed [47] and soybean oils [22] were also included) directly to extract carotenoids. Other eco-friendly solvents included in the selected studies were ethanol, “Eco-Friendly Ethyl Lactate–Ethyl Acetate Solvent”, “hydrophobic eutectic solvents” and “saturated sodium chloride in water”. Apart from that, non-eco-friendly solvents such as hexane, acetone, tetrahydrofuran (THF), butylated hydroxytoluene (BHT), methanol, and acetonitrile were included in the selected articles. 

## 5. Conclusions and Future Perspectives

Since there are more scientific studies related to the extraction of carotenoids from fruits and vegetable by-products than those included in this review, we have just included those containing an RSM for process optimization. Considering the three green extraction technologies included in this review, more than 70% of the evidence is focused on ultrasound-assisted technology. Therefore, there is still room for optimization, while combinations with other green technologies (i.e., pre-enzymatic treatment) could be of interest. Among the selected manuscripts, tomato, followed by citrus, and peach palm by-products were the most used raw materials for carotenoid extraction optimization. Time, temperature, solid-liquid ratio, and solvent were the most studied inputs. The most used RSM design was CCD, followed by BBD, which are basically presented using response surface plots, followed by contour plots. It is worth mentioning that there was a tendency to use green solvents, including edible vegetal oils.

A valuable lesson learned from this systematic review is the importance of providing clear and comprehensive information in reports when applying RSM. The biggest bias observed in the reviewed works was the lack of essential information, as detailed in the following lines. To assist readers in extracting maximum information, it is essential to include a detailed description of the experimental design, a rationale for selecting a particular design, a specification of input variables and their potential levels, a discussion on the statistical model’s validity, and an explanation of the optimization procedure. Further studies should focus on this issue. Also, it is important to highlight that no data of specifications of the methods used to assess the risk of bias in the selected studies was included, including the principles of open science, specifically data availability, in future scientific manuscripts related to RSM, and revalorization is a point to improve. 

## Figures and Tables

**Table 1:** Questions used as quality criteria.

Q1	Does the article include an experimental design based on response surface methodology?
Q2	Does the article explain the reason for the experimental design selection?
Q3	Does the article define input factors and output responses?
Q4	Does the article include factor levels?
Q5	Does the article describe how the factor levels were selected?
Q6	Do the authors report a transformation of the response data?
Q7	Does the article include the number of experiments?
Q8	Does the article show the experimental runs?
Q9	Does the article show the measure of response in graphs?
Q10	Are the data available?
Q11	Are indicators for the quality of fit of the response surface included?
Q12	Is a discussion about the significance of each factor’s effect included?
Q13	Does the paper mention whether the statistical hypothesis of the model was checked?
Q14	Does the article include the process optimization?
Q15	If an optimum is found, was a further experiment carried out to validate the expected results?
Q16	Is the code for data analysis available?

**Table 3:** RSM design, number of levels, and centers (axial points when specified).

Tech.	Study	RSM Design	Levels	Experiments	Centers	Axial Points	Graphical Presentation	Ref.
USAE	**S1**	FD: FrFD (SD) *	5	20	No data	-	Response surface plots and PCA	[27]
	**S2**	CCD	5	30	6	-	Response surface plots	[28]
	**S3**	BBD	3	15	3	-	Response surface plots	[29]
	**S4**	CCD	5	16	2	-	Response surface plots	[22]
	**S5**	CCD: rotatable	5	20	6	-	Response surface plots	[30]
	**S6**	CCD	3	20	6	-	Pareto chart	[31]
	**S7**	CCD	5	29	4	-	Contour plots and response surface plots	[32]
	**S8**	CCD	5	No data	No data	-	Response surface plots	[33]
	**S9**	FD: FrFD	3	9	No data	-	Response surface plots	[34]
	**S10**	CCD: rotatable	5	18	4	6	Contour plots	[35]
	**S11a**	FD: FrFD (SD)	3	19	3	-	Normal graph	[36]
	**S11b**	CCD	3	11	3	-	Contour plots and response surface plots	[36]
	**S12**	BBD	3	15	3	-	Contour plots and response surface plots	[37]
	**S13**	CCD	3	20	6	-	Response surface plots	[38]
	**S14**	BBD	3	29	5	-	Response surface plots	[39]
	**S15**	CCD	5	20	6	-	Response surface plots	[40]
	**S16**	BBD	3	30	6	-	Response surface plots	[41]
	**S17**	BBD	3	30	6	-	Response surface plots	[42]
	**S18**	BBD	3	17	5	-	Response surface plots	[43]
	**S19**	CCD: Face-centred	3	20	6	-	Response surface plots	[44]
	**S20**	CCD	5	17	4	-	Response surface plots	[45]
	**S21**	BBD	3	15	4	-	Response surface plots	[46]
MWAE	**S22**	CCD: rotatable	5	20	6	6	Response surface plots	[47]
	**S23a**	BBD	3	27	3	-	Response surface plots	[48]
	**S23b**	CCD	3	10	2	-	Response surface plots	[48]
	**S24a**	EXP2. FD: FrFD (HFD)	3	8	3	-	Pareto chart	[23]
	**S24b**	EXP3. CCD: rotatable	5	19	6	6	Response surface plots	[23]
	**S25**	BBD	3	18	6	-	Response surface plots	[24]
EAE	**S26**	CCD	5	31	No data	-	Contour plots and response surface plots	[49]
	**S27**	FD: FFD	3	10	No data	-	Response surface plots	[25]
	**S28**	CCD	5	31	7	-	Contour plots and response surface plots	[26]
	**S29**	BBD	3	15	3	-	Response surface plots and PCA	[50]

* CCD: central composite design; BBD: Box–Behnken design; FD: factorial design; FFD: full factorial design; FrFD: fractional factorial design; FrFD (SD): fractional factorial design (screening design); HFD: half-fractional factorial design.

**Table 4:** Number of inputs, specific inputs (I1–I19), and number of responses in each of the selected studies included in this paper.

Tech.	Study	Inputs	I1 *	I2	I3	I4	I5	I6	I7	I8	I9	I10	I11	I12	I13	I14	I15	I16	I17	I18	I19	Responses	Ref.
USAE	**S1**	4																				5	[27]
	**S2**	3																				1	[28]
	**S3**	3																				2	[29]
	**S4**	3																				1	[22]
	**S5**	3																				1	[30]
	**S6**	3																				1	[31]
	**S7**	4																				3	[32]
	**S8**	2																				1	[33]
	**S9**	3																				1	[34]
	**S10**	3																				2	[35]
	**S11a**	5																				1	[36]
	**S11b**	3																				1	[36]
	**S12**	3																				1	[37]
	**S13**	3																				1	[38]
	**S14**	4																				1	[39]
	**S15**	3																				10	[40]
	**S16**	4																				1	[41]
	**S17**	4																				1	[42]
	**S18**	3																				4	[43]
	**S19**	3																				3	[44]
	**S20**	3																				4	[45]
	**S21**	3																				3	[46]
MWAE	**S22**	3																				1	[47]
	**S23a**	4																				5	[48]
	**S23b**	2																				5	[48]
	**S24a**	4																				4	[23]
	**S24b**	3																				4	[23]
	**S25**	3																				4	[24]
EAE	**S26**	4																				2	[49]
	**S27**	2																				1	[25]
	**S28**	4																				1	[26]
	**S29**	3																				1	[50]

* I1: time; I2: temperature; I3: ratio solid:liquid; I4: solvent; I5: amplitude; I6: concentration of enzyme; I7: number of extractions; I8: microwave power; I9: ultrasound intensity; I10: power; I11: saponification time; I12: saponification solution concentration; I13: extraction technique; I14: ionic reagent in the solvent; I15: pulse cycle; I16: energy equivalents; I17: microwave power amplitude; I18: pH; and I19: stirring speed.

## Data Availability

Data sharing is not applicable—no new data are generated.

## Supplementary Materials

The following supporting information can be downloaded at: https://www.mdpi.com/article/10.3390/foods12244456/s1, Table S1: PRISMA 2020 Checklist.
